# Glucocorticoids promote the development of azoxymethane and dextran sulfate sodium-induced colorectal carcinoma in mice

**DOI:** 10.1186/s12885-019-5299-8

**Published:** 2019-01-21

**Authors:** Bo Li, Yan Wang, Lijuan Yin, Gaoxiang Huang, Yi Xu, Jie Su, Liye Ma, Jian Lu

**Affiliations:** 1 Department of pathophysiology, Second Military Medical University, 800 Xiangyin Road, Shanghai, 200433 People’s Republic of China; 2 Department of general surgery, Changhai hospital, Second Military Medical University, 168 Changhai Road, Shanghai, 200433 People’s Republic of China; 3 Department of pathology, Changhai hospital, Second Military Medical University, 168 Changhai Road, Shanghai, 200433 People’s Republic of China

**Keywords:** Corticosterone, Azoxymethane/dextran sodium sulfate, Colorectal carcinoma, Tumor development, Nuclear factor-κB

## Abstract

**Background:**

Stress has been suggested as a promoter of tumor growth and development. Glucocorticoids (GCs) are the main stress hormones and widely prescribed as drugs. However, the effect of GCs on the development and progression of colorectal carcinoma (CRC) is unclear.

**Methods:**

We evaluated the effect of corticosterone (CORT) on azoxymethane and dextran sulfate sodium (AOM/DSS)-induced carcinogenesis in the colorectum of C57BL/6 strain mice. Plasma level of CORT was detected by radioimmunoassay. The expression of proliferation markers (Ki-67 and PCNA), nuclear factor (NF)-κB p65 and phosphoto-p65 (P-p65), as well as cyclooxygenase (COX)-2 were determined by immunohistochemistry. Inflammation in colorectum was evaluated by histopathology.

**Results:**

CORT feeding in drinking water of mice not only significantly elevated plasma CORT concentration, but also significantly increased the incidence and neoplasms burden (number and size of neoplasms) in colorectum. CORT also significant enhanced the expression of cell proliferation marker (Ki-67 and PCNA), NF-κB p65 and P-p65 as well as COX-2 in colorectal neoplasm of AOM/DSS-treated mice.

**Conclusion:**

In this study, we have found for the first time that CORT at stress level potentially promotes the growth and development of AOM/DSS-induced colorectal adenoma and carcinoma in mice. Up-regulation of NF-κB and COX-2 may be involved in the promoting effect of CORT.

## Background

Clinical studies have linked the experience of stressful events, such as pressure, cancer-related concerns and depression, to poor survival of cancer patients [1, 2]. Preclinical studies also support that chronic stress has an impact on cancer progression and survival [3–5]. Glucocorticoids (GCs) are main stress hormones which are secreted dramatically in a state of stress, and play a critical role in the process of immunosuppression, anti-inflammation and homeostasis sustaining [6]. Synthetic GCs, such as dexamethasone, have been widely used as drugs to treat immune and inflammatory disorders. Moreover, GCs are clinically important as adjuvants in non-hematologic cancer therapy to reduce acute toxicity and alleviate side effects induced by chemotherapy or radiotherapy [7]. GCs exert their biological effects by regulating the expression of genes and cross-talking with multiple trans-membrane signalling pathways [8]. The effects of GCs are mediated by glucocorticoid receptor (GR), which is ubiquitously expressed in all cells. Since the activation of GR by GCs control a variety of physiological and cellular processes, such as immune response, metabolism, cell proliferation, apoptosis and survival [9], the relationship between GCs and solid tumors has been concerned. Although there are reports that GCs have the inhibitory effect on proliferation and progression in several tumors in vitro and in vivo [10–13], other and our studies have demonstrated that GCs induce resistance to chemotherapy in the majority of solid tumor cells [14–16], promote metastasis of pancreatic cancer [17] and melanoma cells [18], and increase risks of skin cancer and non-Hodgkin’s lymphoma [19]. It still remains unclear whether these controversial phenomena are attributed to the dose and duration of GC treatment, as well as cell and tissue specific GC signal transduction. Therefore, it is vital to further evaluate the role of GCs in carcinogenesis and progression of different solid tumors, especially in vivo.

Colorectal carcinoma (CRC) is one of the top three diagnosed cancers worldwide. It can develop spontaneously or as a complication of inflammatory bowel diseases. It is believed that chronic inflammation increase the risk of CRC development [20]. Limited studies about the effect of GCs on growth of CRC have been done in vitro and in vivo, and the results are not concordant. Inhibitory effect or promoting effect that GCs showed on growth of CRC depended on cell lines [21–23], tumor micro-environment [24, 25] and the delivery formulation and dosage of GCs [26]. In addition, previous animal studies were done using the xenografts model mice which are featured by immunodeficiency, while the result from these mice is not equal to that of intact mice with induced carcinoma in colorectum spontaneously. A mouse CRC model induced by azoxymethane and dextran sulfate sodium (AOM/DSS) is a stable and reliable model of spontaneous tumor and has been used in CRC studies [27–29]. AOM is a chemical mutagen, which induces colonic tumor with clinical, histological and molecular features of human sporadic colon cancer, and the follow-up treatment with the non-genotoxic DSS strongly induces inflammation in the colon, thereby enhancing the colon carcinogenesis in rodents [30]. In this study, we investigated whether corticosterone (CORT), a major form of GCs in mouse, influenced the development of CRC in such a mouse model of colorectal carcinogenesis induced by AOM/DSS.

## Methods

### Animals

All animal experiments were approved by the Animal Ethics Committee of Second Military Medical University (Shanghai, China) and performed in compliance with the University’s Guidelines for the Care and Use of Laboratory Animals. Six to 8-week-old male mice of C57BL/6 strain (body weight 20 ± 2 g) were purchased from Model Animals Research Centre of Nanjing University (Nanjing, China) and housed under a 12-h light/dark cycle in a specific-pathogen-free (SPF) condition with free access to laboratory chow and water in Experimental Animals Centre of Second Military Medical University. Every cage housed no more than 5 mice. All the mice were fed under the above condition for 2 weeks before the experiment to make the mice adapt to the new environment.

### Induction of colorectal carcinogenesis (AOM/DSS treatments)

**Fig. 1:**
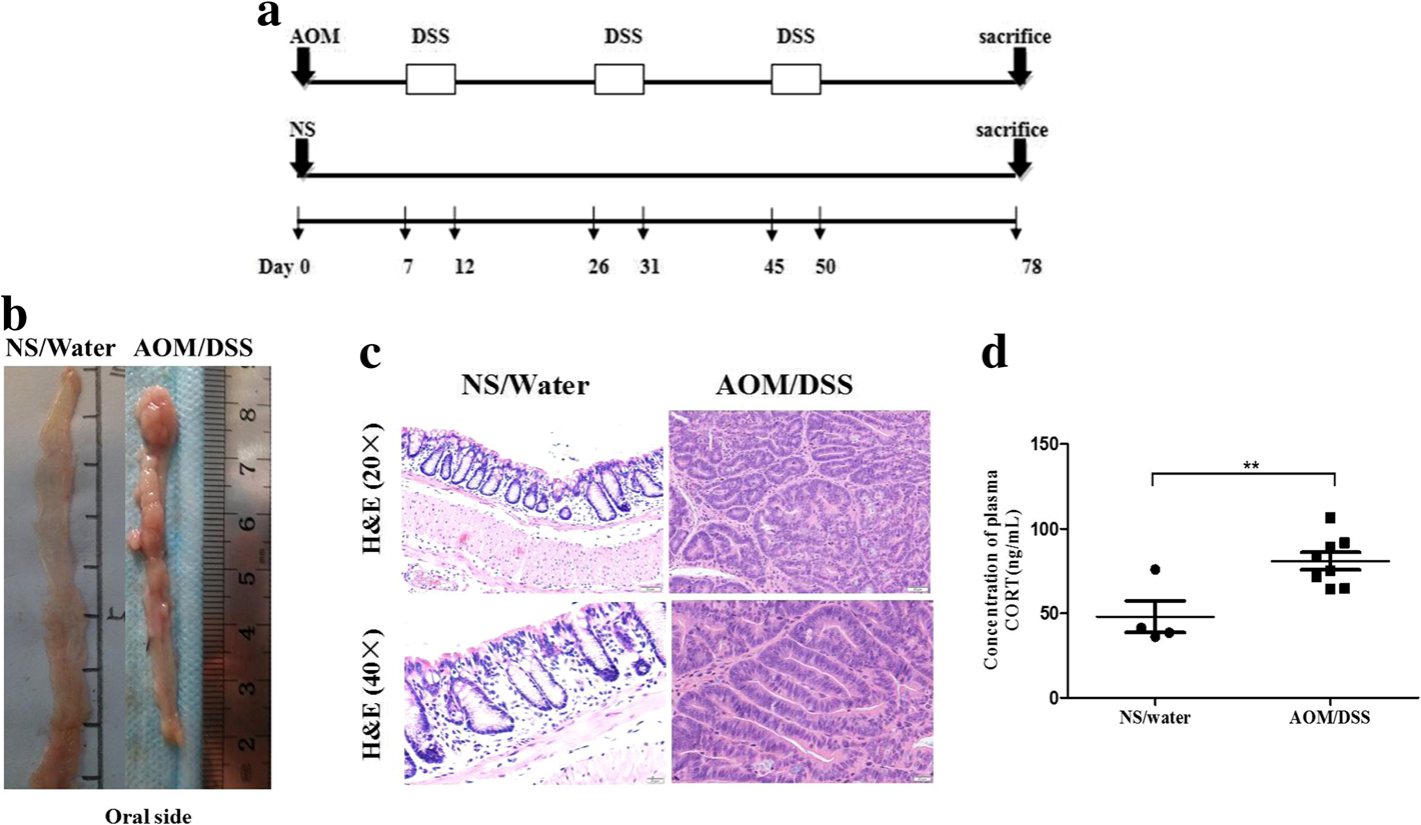
AOM/DSS treatment induces CRC in C57BL/6 male mice successfully and increases the plasma CORT of model mice. The scheme shows one time administration of AOM followed by three cycles of DSS treament and separated by 14-day recovery periods. Colon was examined 28 days after the third cycle of DSS treatment (**a**). Representative longitudinal views of colorectum from control (NS/Water) mice and model (AOM/DSS) mice (**b**). Pathological examination confirmed the most neoplasms of model mice as adenocarcinoma (**c**). Plasma CORT of model mice (number = 8) and control mice (number = 4) were tested by radioimmunoassay (**d**). Data expressed as mean ± SD. **P < 0.01 by unpaired *t*-test analysis. *AOM* azoxymethane,* DSS* dextran sodium sulphate, *CRC* colorectal carcinoma, *NS* normal saline, *CORT* corticosterone

We followed an outlined protocol [27] and made some modification. Mice (number = 10) were injected intraperitoneally with 12.5 mg/kg body weight of AOM (Sigma) dissolved in normal saline (NS) and maintained on a regular chow and water. After 7 days, the mice were fed with 2.5% DSS (MW 36000–50,000 kDa, Biomedicals) in the drinking water for 5 days, followed by 14 days of regular drinking water for recovery. This cycle was repeated 3 times, after that, mice were fed with regular chow and water till the end (Day 78). Control mice (number = 4) were injected intraperitoneally with NS on the first day and given with free access to laboratory chow and water till the end that the mice were killed by cervical dislocation (Fig. 1a). Two mouse in AOM/DSS treatment group and no mice in control group died before the end of experiment.

### CORT treatments

**Fig. 2:**
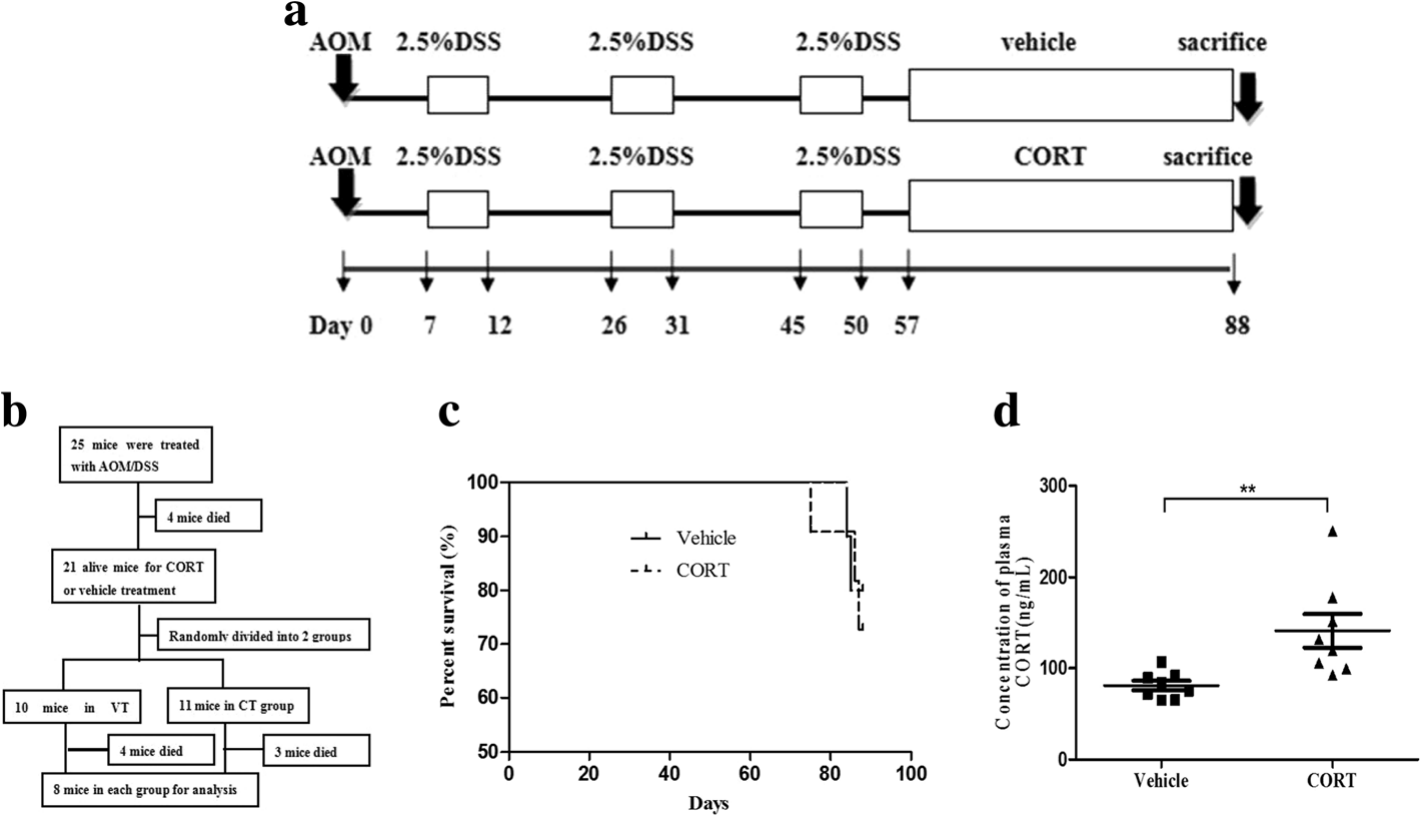
CORT feeding increases plasma CORT of AOM/DSS-induced CRC mice. The scheme shows model mice recovered for 7 days after the last cycle of DSS treatment and then undertook with vehicle treatment (VT, 0.5% ethanol, number = 10) or CORT treatment (CT,50 mg/L, number = 11) randomly for 31 days (**a**). Methodology workflow of the study (**b**). Survival rate of CT mice (number = 11) and VT mice (number = 10) (**c**). Plasma CORT of CT mice (number = 8) compared with VT mice (number = 8) (**d**). Data expressed as mean ± SD. *P < 0.05, **P < 0.01 by unpaired *t*-test analysis. *CORT* corticosterone, *CT* CORT treatment, *VT* vehicle treatment

CORT (Sigma) was dissolved in ethanol to obtain the working solution (10 mg/mL), which was then diluted with water to get the feeding solution (50 mg/L) according to the literature [31–33]. As the overall experiment schedule showed in Fig. 2a, model mice recovered for 7 days after the last cycle of DSS treatment and then fed with vehicle (0.5% ethanol, number = 10) or CORT (number = 11) randomly for 31 days till killed by cervical dislocation for further analysis. Two mice in vehicle group and 3 mice in CORT group died before the end of experiment. Figure 2b outlines the methodology workflow of the study.

### Quantification of CORT in vivo

At the end of the experiment, all mice were anesthetized with 5% ethyl urethane (30 μL/g body weight) for blood collection from the ciliary venous plexus at dusk to avoid the influence of physiological GC fluctuation in vivo as far as possible. Collected blood samples were placed at 4 °C for 20 min and then centrifuged at 4 °C for 1 min at 10000 rpm, and the liquid supernatants were used for CORT detection by radioimmunoassay and expressed as pg/mL plasma.

### Macroscopic observation

Mice were weighed weekly during the whole experiment. At the end of the experiment, all mice were killed by cervical dislocation and then dissected. Colorectum was removed, opened longitudinally and rinsed with ice-cold phosphate buffer solution for macroscopic inspection, and then fixed overnight with 4% formalin for histological analysis. Every entire colorectum was measured in length and photographed. Grossly visible neoplasms were counted and measured in diameter with an electronic vernier caliper. The sums of area load and volume load per colorectum were calculated with the formula, area = [(length + width)*0.5]^2^ and volume = length*width^2^*0.52. Evaluation was performed without any knowledge of the treatment type.

### Histopathology, inflammation score analysis and immunohistochemistry analysis

Colorectal tissues were processed by routine histological methods: fixed, sampled, dehydrated, embedded in paraffin, sectioned and stained with hematoxylin and eosin (H&E) for histopathological evaluation, which were preformed independently by two trained pathologists in a blinded manner. According to histopathology, adenoma with low grade dysplasia was defined as adenomatous changes with simple glandular architecture and low grade nuclei form. Adenoma with high grade dysplasia was defined as adenomatous changes with architectural complexities and high grade nuclei form. Intramucosal carcinoma was defined as high-grade dysplasia with invasion of neoplastic cells into the lamina propria but not through the muscularis mucosa. Adenocarcinoma (ACA) was defined as invasion of neoplastic cells into or even through the submucosa [34, 35].

Histological evaluation of inflammation in the colorectum was performed using a semi-quantitative scoring system by two pathologists. In brief, scores were given for percent ulceration (0–3), percent reepithelialization (0–4), active inflammation (0–3), chronic inflammation (0–3), and transmural inflammation (0–3) [36]. Inflammation was scored as follows: 0, no inflammation; 1, modest numbers of infiltrating leukocytes (neutrophile granulocyte for acute inflammation and lymphocyte for chronic inflammation) in the lamina propria; 2, infiltration of leukocytes leading to separation of crypts and mild mucosal hyperplasia; 3, massive infiltration of inflammatory cells accompanied by disrupted mucosal architecture and complete loss of goblet cells. Then sum the scores up for analysis. The tissues embedded in paraffin were cut serially into 4-μm-thick sections for immunohistochemistry (IHC) analysis. IHC staining with rabbit anti-mouse proliferating cell nuclear antigen (PCNA) polyclonal antibody (dilution 1:200, Santa cruz, #sc-7907), rabbit anti-mouse Ki-67 monoclonal antibody (dilution 1:200, abcam, #ab16667), rabbit anti-mouse NF-κB p65 monoclonal antibody (dilution 1:100, CST, #4764), rabbit anti-mouse NF-κB P-p65 monoclonal antibody (dilution 1:100, Ser536, CST, #3033) and rabbit anti-mouse COX-2 monoclonal antibody (dilution 1:100, cayman chemical, #160112) were performed using EnVision™ systems (Dako, Demark) according to the manufacturer’s instructions.

### Statistical analysis

Statistical analysis was performed using GraphPad Prism (version 5.0) software or SPSS (version 18.0) software. Data were expressed as the means ± SD. The unpaired t-test (Student’s t-test) was used to compare with two groups, and the one-way or two-way ANOVA was used to compare with more than two groups. A Pearson’s χ^2^ test was used to test the significance of association between different treatment and pathological diagnosed types. Differences were considered significant at *P* < 0.05.

## Results

### Establishment of colorectal carcinoma murine model

Firstly, we established the mice CRC model according to a modified protocol for the AOM/DSS-induced colorectal cancer (Fig. 1a). At the end of the experiment, mice were sacrificed for further analysis. Macroscopic observation of colorectum showed that the length of colorectum of model mouse was much shorter than that of the control one. Neoplasms were observed in the colons and recta of the model mice, but not in the control ones (Fig. 1b). Pathological examination confirmed the most of neoplasms as adenocarcinoma (ACA) (Fig. 1c). Meanwhile, we tested the plasma CORT of mice by radioimmunoassay and the results showed that the plasma levels of CORT in the model mice were higher than those of the control ones (1.68-fold, *P* < 0.01, Fig. 1d), which may be due to tumor-related stress induced by AOM/DSS.

### CORT treatment significantly increased the plasma CORT concentration

Furthermore, we administrated the model mice with CORT treatment (CT) or vehicle treatment (VT). CORT administration can be performed in different ways, such as implanting extended-release agents subcutaneously, intraperitoneal injection or feeding in drinking water, at different stages of CRC model induced by AOM/DSS. We firstly fed CORT (50 mg/L) in drinking water or implanted extended-release CORT (7.5 mg-60 days) subcutaneously at the early stage (after the first cycle of DSS) of model mice. However, both ways of CORT administration led to high mortality of model mice, which may ascribe to high metabolism rate and immunosuppression of CORT. So we fumbled experimental condition and put off the time of CORT administration to a week after the third cycle of DSS treatment. Compared with implanting extended-release CORT agents which may cause stress in the implanting surgery, we chose CORT administration in drinking water (Fig. 2a). Treating mice with CORT for 31 days in this way did not significantly affect the death rate (3/11 of CT versus 2/10 of VT) of mice (Fig. 2c). At the end of experiment, we found that plasma CORT level of CT mice further increased about 1.74-fold compared with that of VT ones (*P* < 0.01, Fig. 2d). We also observed more rich of omental fat in the CT mice compared with that of VT ones and it indicated that CORT slightly increased the body weight of mice, which may be due to the fat storage effect of CORT (data not shown).

### CORT promoted development of AOM/DSS-induced colorectal carcinoma in mice

**Fig. 3:**
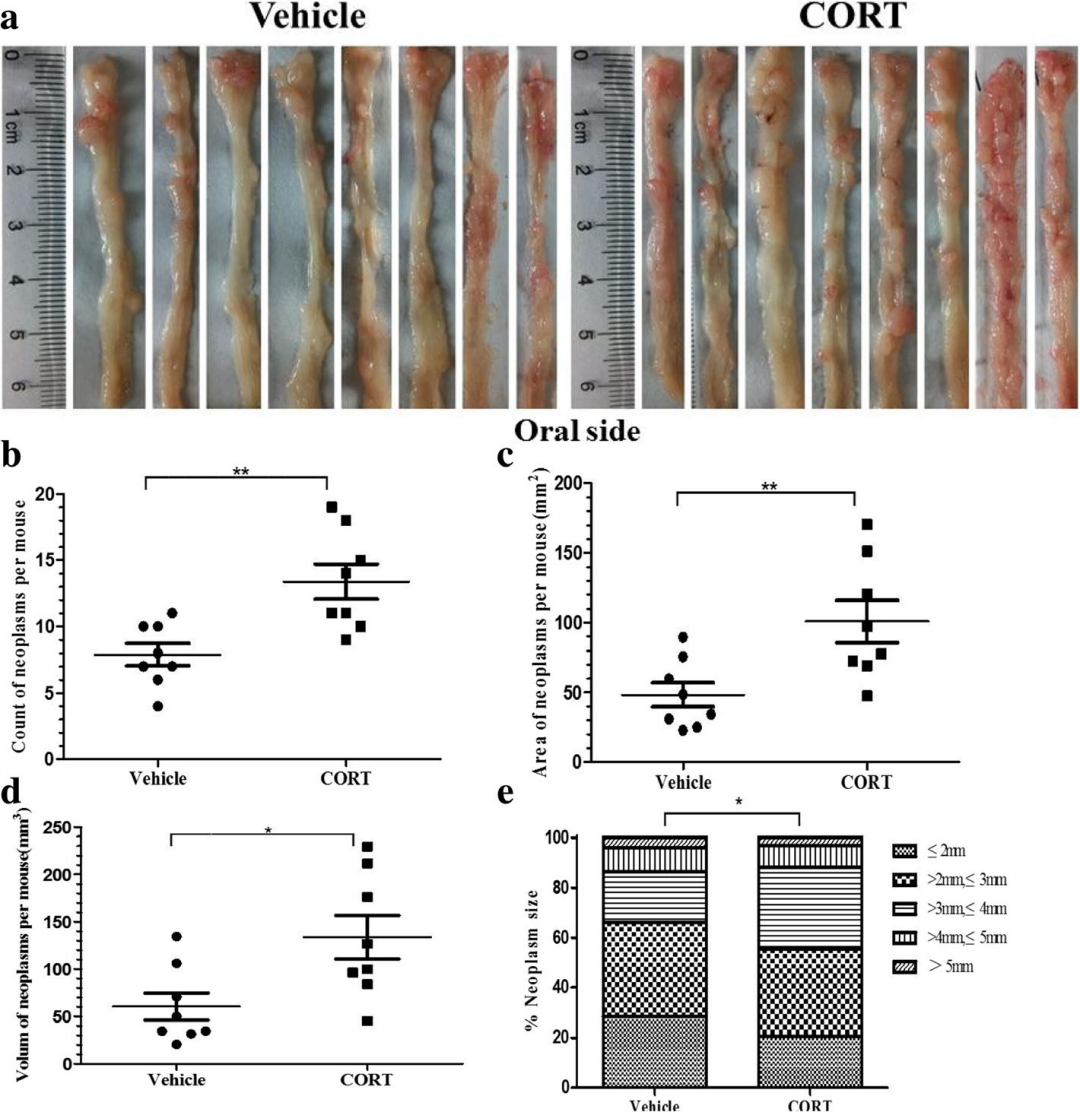
CORT feeding increases development of AOM/DSS-induced CRC in mice. Representative longitudinal views of colorectum from the VT mice and the CT mice (**a**). Count of neoplasms per mouse (**b**), area of neoplasms per mouse (**c**) and volume of neoplasms per mouse (**d**) of the VT and the CT mice. Percentage of neoplasms per size group for each treatment mice (**e**). Number = 8 per group. Data expressed as mean ± SD. *P < 0.05, **P < 0.01 by unpaired t-test and two-way ANOVA for neoplasms size distribution analysis. Length and width of neoplasms were measured by electronic vernier caliper. Area = [(length + width)*0.5]^2^, Volume = length*width^2^*0.52. *CT* CORT treatment, *VT* vehicle treatment

Then we investigated the effects of CORT on the development of AOM/DSS-induced CRC in mice. Macroscopic views of the colorectum from each treatment mice are shown in Fig. 3a. A higher neoplasm burden was observed especially in the distal colon and rectum areas of the CT mouse as compared with that of the VT one. Further analysis showed that administration of CORT resulted in significantly increased number (1.7-fold, *P <* 0.01), area (2.1-fold, *P* < 0.01) and volume(2.2-fold, *P* < 0.05) of neoplasms in colorectum (Fig. 3b, c and d). The size distribution of neoplasms in CT mice was obviously larger than that of the VT ones, especially in diameter more than 3 mm (Fig. 3e). Pathological examination showed that main histological alteration of colorectum was adenoma with low grade dysplasia (62.5%) and adenoma with high grade dysplasia (25%) in the VT mice, whereas intramucosal carcinoma or ACA (75%) in the CT mice (Table 1). These results indicated that the administration of CORT significantly increased the total incidence of colorectal neoplasms and promoted the shift of pathological types (from adenoma with low grade dysplasia to intramucosal carcinoma or ACA) in model mice.

**Table 1:** Histological tumor incidence and multiplicity in AOM/DSS-induced mice treated with CORT or vehicle in drinking water

Group	Number	% of microscopic lesions classified as		
		Adenoma with low grade dysplasia	Adenoma with high grade dysplasia	Intramucosal carcinoma &ACA
VT	8	62.5	25	12.5
CT	8	12.5	12.5	75

*ACA* adenocarcinoma, *CT* corticosterone treatment, *VT* vehicle treatment

### Elevated expression of cell proliferation marker in CORT-treated mice

**Fig. 4:**
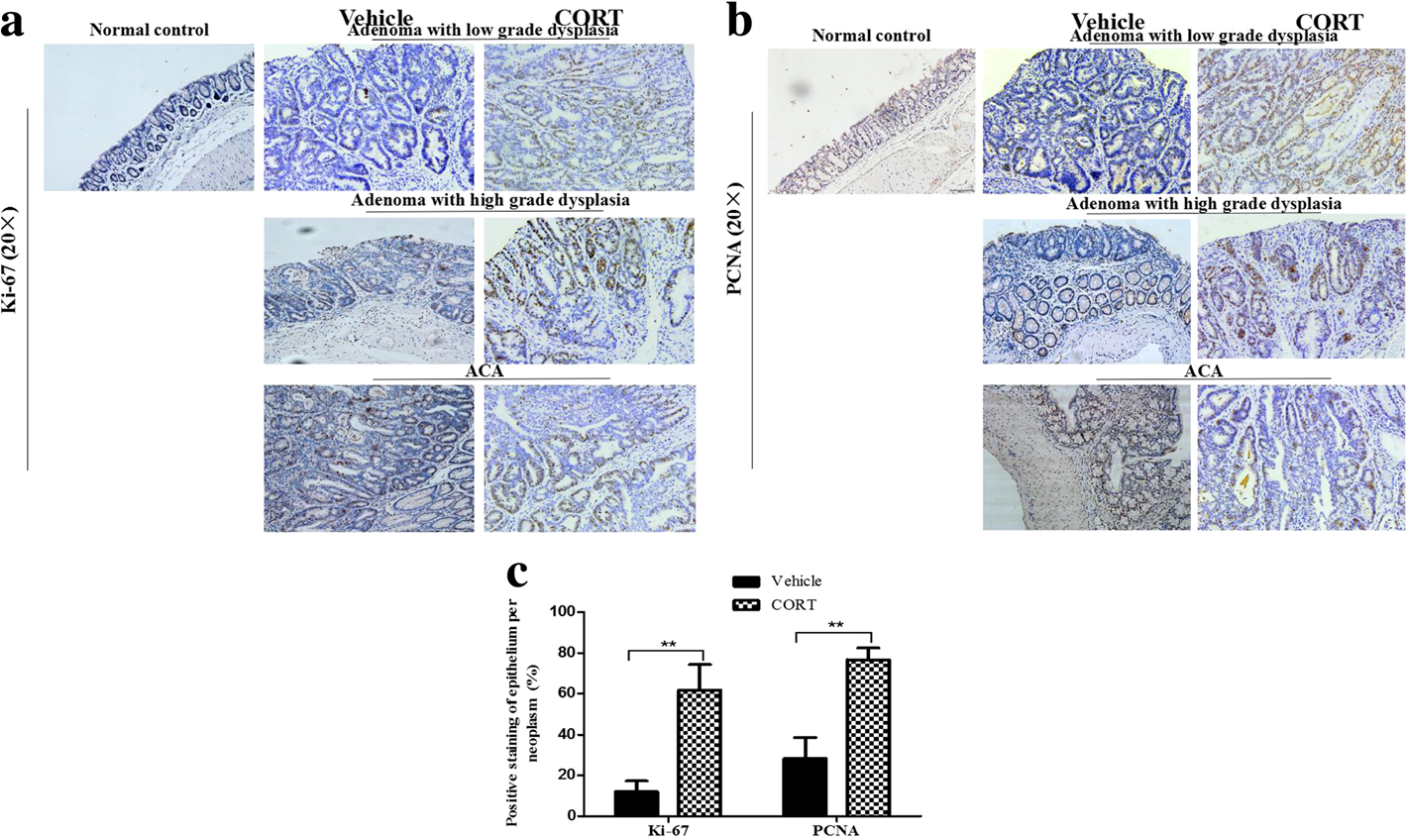
CORT feeding elevates expression of proliferation marker in AOM/DSS-induced CRC mice. The colorectum of model mice with VT or CT (number = 3 per group) were processed for immunostaining of proliferation marker. Representative images of normal colorectal epithelia (Normal control) and neoplasms area, classified pathologically as adenoma with low grade dysplasia, adenoma with high grade dysplasia and ACA, stained with antibody of Ki-67 (**a**, at 20X), PCNA (**b**, at 20X) and their bar diagrams showing the percentages of Ki-67 and PCNA positive colonic epithelial cells (**c**). Data expressed as mean ± SD. **P<0.01 by unpaired *t*-test analysis.* CT* CORT treatment, *VT* vehicle treatment

We further examined the expression of cell proliferation marker, Ki-67 and PCNA in the neoplasms of both CT and VT mice as well as normal colorectal epithelia with IHC analysis. The results showed that administration of CORT significantly elevated Ki-67 and PCNA expression in colonic epitheliums of neoplasms, including pathological type adenoma with low grade dysplasia, adenoma with high grade dysplasia and ACA. Whereas in normal epithelia, Ki-67 is mainly expressed in basal cells, but hardly expressed in glandular epithelial cells, and the staining of PCNA are rarely seen (Fig. 4a and b). Figure 4c showed the statistical results of positive staining percent, which were consistent with the macroscopic observation results, indicating that CORT promoted the growth and development of AOM/DSS-induced colorectal adenoma and carcinoma in mice.

### CORT promoted expression of NF-κB and COX-2 in colorectal neoplasms

**Fig. 5:**
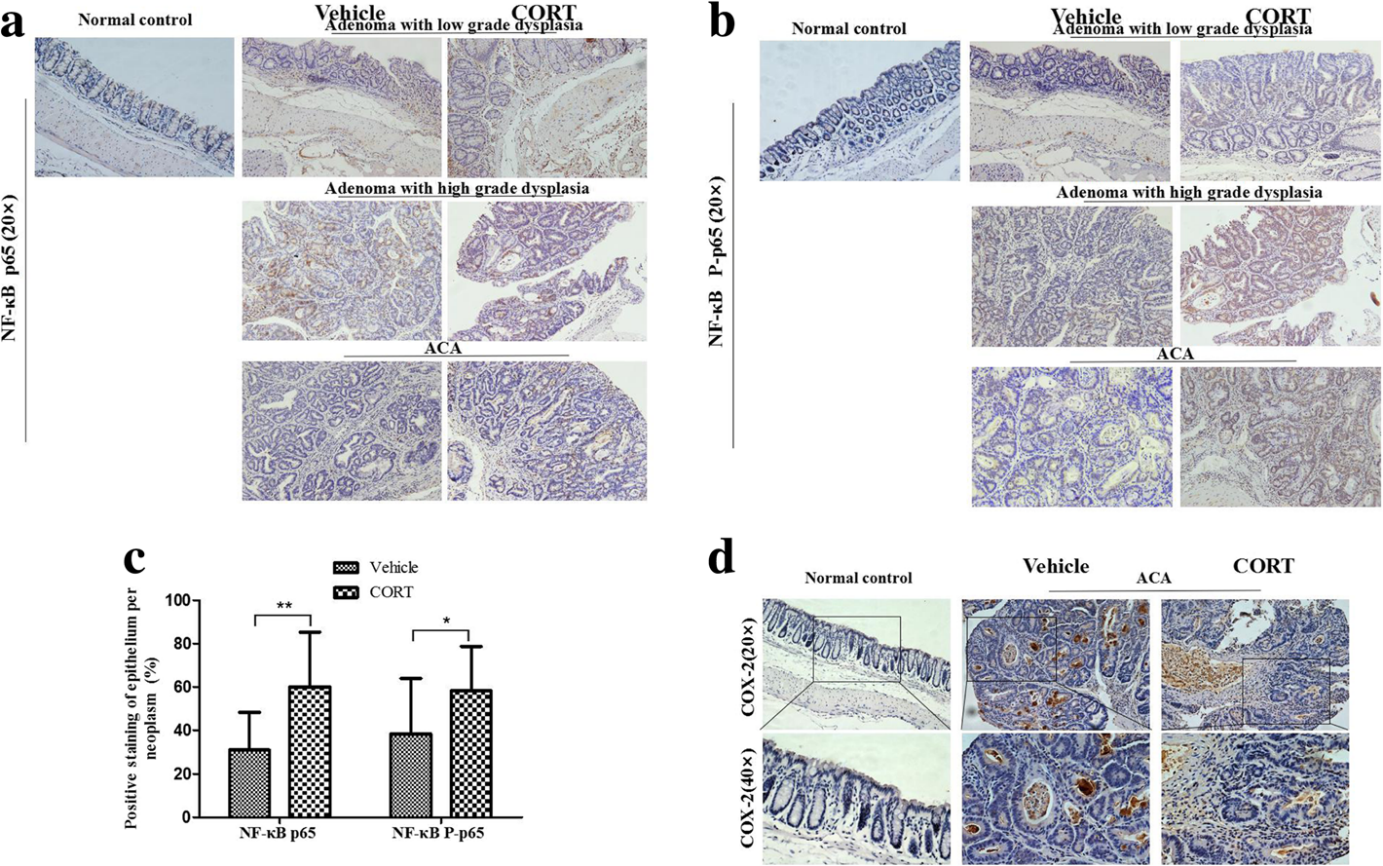
CORT feeding elevates expression of NF-κB and COX-2 in AOM/DSS-induced CRC in mouse. The colons of model mice with CT or VT (number = 6 per group) were processed for immunostaining of NF-κB and COX-2. Representative images of normal colorectal epithelia (Normal control) and neoplasms area, classified pathologically as adenoma with low grade dysplasia, adenoma with high grade dysplasia and ACA, stained with antibody of NF-κB p65 (**a**, at 20X), NF-κB P-p65 (**b**, at 20X) and their bar diagrams showing the percentages of NF-κB p65 and P-p65 (**c**) positive cells of different treatment groups. Representative images of normal colorectal epithelia (Normal control) and ACA area stained with antibody of COX-2 (**d**, at 20X and 40X). Data expressed as mean ± SD.*P<0.05, **P<0.01 by *t*-test for IHC data. *ACA* adenocarcinoma, *IHC* immunohistochemistry, *CT* CORT treatment, *VT* vehicle treatment

Nuclear factor (NF)-κB, which consists of a number of closely related protein dimers (mainly the p50 and p65) is an important transcription factor and a key mediator of immune and inflammatory responses. Phosphorylation of p65(P-p65) by several kinase leads to optimal NF-κB activation. There is growing evidence that NF-κB is essential for promoting inflammation-associated cancer, including CRC, by enhancing cell proliferation and angiogenesis, inhibiting apoptosis, and promoting cell invasion and metastasis [37, 38]. To elucidate the mechanisms of CORT promoting growth in AOM/DSS-induced CRC, we detected the expression of p65 and P-p65 in the colorectal neoplasms, including pathological type adenoma with low grade dysplasia, adenoma with high grade dysplasia and ACA, in VT and CT mice as well as normal colorectal epithelia with IHC analysis. The results showed that p65 and P-p65 expressed in both epithelium cells and stromal cells of neoplasms, but rarely in normal colorectal epithelia (Fig. 5a and b). More P-p65 was observed in the nuclei than cytoplasm. The expression of p65 and P-p65 in the colorectal cells of the CT mice significantly increased by 94.8 and 52.2% respectively compared with that of the VT ones (Fig. 5c). When activated NF-κB translocates to the nucleus, it can regulate the expression of multiple target genes, including COX-2. It is widely accepted that the deregulation of the COX-2 signalling pathway took a vital effect in the progression of CRC [39]. Therefore, we investigated the expression of COX-2 by IHC analysis. The result showed that COX-2 mainly expressed in stromal cells, which mainly may be lymphocyte featured as round shape and fibroblast featured as fusiformis shape, of colorectal neoplasms, which were consistent with previous reports [40]. Administration of CORT also resulted in increased expression of COX-2 in stromal cells of the neoplasms compared with those of the VT mice (Fig. 5d).

### CORT inhibit the inflammation of neoplasms in AOM/DSS-treated mice

**Fig. 6:**
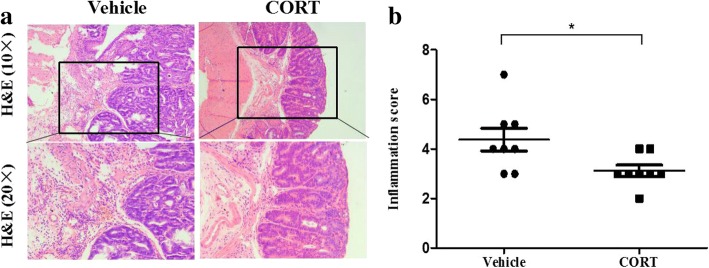
CORT feeding inhibits inflammation of neoplasms from AOM/DSS-induced CRC mice. The colorectum of model mice with CT or VT (number = 8 per group) were processed for H&E staining. Representative images of neoplasm area (**a**, at 10X and 20X). Semi-quantitative histological measurement of total inflammation (inflammation score) were calculated by adding the values for percent ulceration, percent re-epithelialization, and active, chronic and transmural inflammation (**b**). Data expressed as mean ± SD. *P<0.05 by unpaired *t*-test analysis. *H&E* hematoxylin and eosin, *CORT* corticosterone, *CT* CORT treatment, *VT* vehicle treatment

In this study we used a mouse model of colorectal carcinogenesis induced by AOM/DSS. DSS strongly induces inflammation in the colon, thereby enhancing the colon carcinogenesis in mice [30]. While GC has an anti-inflammatory effect, therefore, we histologically evaluated inflammation in the stromal tissue of neoplasms (Fig. 6a) of both treatment mice at the end of the experiment. The inflammation scores (including ulceration, reepithelialization, infiltration of inflammatory cells and transmural inflammation) were evaluated using a semi-quantitative scoring system by two pathologists. As shown in Fig. 6b, the inflammation score of the CT mice was significantly lower than that of the VT ones (*P* = 0.0288), indicating that CORT treatment alleviates inflammation in the colon of AOM/DSS induced model mice.

## Discussion

GCs are main stress hormones and commonly prescribed in clinical practice. Although it is well known that they control a variety of physiological and cellular processes, such as immune response, metabolism, cell proliferation, cell apoptosis and cell survival [9], the effect of GCs on the development and progression of CRC is unclear. Therefore, in this study we examined the association between CORT at stress level and development of CRC with an AOM/DSS-induced mice model. We showed that the plasma levels of CORT in model mice were significantly higher than that of control mice, which may be due to tumor-related stress caused by AOM/DSS. CORT feeding in drinking water after the third cycle of DSS treatment further elevated the plasma levels of CORT. Increased CORT not only significantly enhanced the neoplasms burden (number and size), but also increased incidence of adenoma with high grade dysplasia and ACA in pathological type. Furthermore, CORT obviously enhanced the expression of two proliferation marker, Ki-67 and PCNA. These results indicated that CORT promoted the growth and development of colorectal adenoma and carcinoma in an induced mice CRC model by AOM/DSS spontaneously.

It is well known that as a transcription factors, activated NF-κB migrates into the nucleus to regulate the expression of multiple target genes involved in the development and progression of cancer [37]. In CRC, NF-κB has a key role in cancer-related processes, including enhancing cell proliferation and angiogenesis, inhibiting apoptosis and promoting cell invasion and metastasis. Although it is known that GR exerts anti-inflammatory action in part by antagonizing NF-κB, there is also report that stress-related pathophysiological concentrations of GCs increase DNA binding activity of NF-κB (p65 and p50) and production of proinflammatory cytokines, such as IL-6, therefore promoting growth of some type of cancer cells [41–43]. In order to elucidate the mechanism of the above finding, we detected the expression of NF-κB p65 and its active form P-p65, and found that CORT significantly increased NF-κB expression, including the activated form, in the colorectal neoplasms. Moreover, CORT also increased the expression of COX-2, a target gene of NF-κB, in stromal cells of colorectal neoplasms. COX enzymes produce a number of substances, including prostaglandins, which function as major effectors of cancer initiation and progression [20]. Previous study reported that increased COX-2 expression in the tumor environment is associated with poor prognosis in CRC patients [44]. So we suggested that NF-κB-COX-2 pathway may be contributed to growth and development of CRC.

In this study we used a mouse model of colorectal carcinogenesis induced by AOM/DSS. DSS can strongly induce inflammation in the colon, thereby enhancing the colon carcinogenesis in mice [30]. While GC actions typically are described as anti-inflammatory effect, therefore we histologically evaluated the inflammation of neoplasms in the colorectum of mouse. The result showed that the inflammation score of neoplasms in CORT-treated mice were decreased as compared with those of vehicle-treated mice. Why CORT inhibits inflammation but promotes the growth and development of CRC is not known yet. The reason may be that the effect of GC on tumor in vivo is very complex. Besides effects of immunosuppression and anti-inflammation, GC also affects tumor micro-environment and biology of tumor cell. Recently other and our studies demonstrated that GC can directly promote tumor cell survival, proliferation and metastasis by regulating gene expression and activating multiple trans-membrane signalling pathways, such as PI-3 K-AKT pathway [15, 18], TGFβ pathway [17], NF-κB-IL-6 pathway and JNK/AP-1 pathway [17]. Whereas, these effects of GC are not related to inflammation. Another reason may be due to duration of COTR treatment. A significant body of evidence indicates that enhancing inflammation especially in the early stage of colitis promoted colonic tumorigenesis in AOM/DSS-induced mice [45]. Whereas in this study we treated mice with CORT at the late stage (a week after the last cycle of DSS treatment). Perhaps in the late stage the promoting effect of GCs on tumor is more obvious than the effect of inflammation on tumor. The second question is why CORT activates NF-κB and up-regulates the expression of COX-2, but decreases the inflammation score. Although NF-κB and COX-2 are important inflammatory factors, researches in the past years have unveiled that GCs/GR play anti-inflammatory role through complex mechanisms [46], including inhibiting activities of various transcription factors, such as AP-1, cAMP response element-binding protein (CREB) besides NF-κB, inducing several anti-inflammatory protein, such as annexin 1 and MAP kinase phosphatase 1 (MKP1 or DUSP1), also inducing apoptosis of inflammatory cells. So we suggested that anti-inflammatory effect of CORT in our study may through other anti-inflammatory mechanism than NF-κB -COX-2 pathway, which may have a role in promoting development of AOM/DSS induced colorectal carcinoma. GC actions in vivo are more complex than previously anticipated. It is unclear how CORT promotes the development of CRC in this mice model now. Further studies need to be done to clarify the mechanism of GC effect on CRC.

## Conclusions

In conclusion, the present findings show that CORT at stress level dramatically promotes the growth and development of AOM/DSS-induced colorectal adenoma and carcinoma in mice for the first time. The increased expression of NF-κB and its target COX-2 may be involved in CORT effect of promoting development of CRC. These new data provide a potential explanation that chronic stress may exert an important impact on CRC progression by the increasing of GCs level, and remind us to pay attention to the disadvantage of GCs for CRC patients.

## Abbreviations

ACA: Adenocarcinoma
AOM: Azoxymethane
CORT: Corticosterone
COX: Cyclooxygenase
CRC: Colorectal carcinoma
CT: Corticosterone treatment
DSS: Dextran sodium sulphate
GC: Glucocorticoid
GR: Glucocorticoid receptor
H&E: Hematoxylin and eosin
IHC: Immunohistochemistry
NF: Nuclear factor
NS: Normal saline
PBS: Phosphate buffer solution
PCNA: Proliferating cell nuclear antigen
SPF: Specific- pathogen-free
VT: Vehicle treatment
